# The Role of Maxillofacial Structure on Condylar Displacement in Maximum Intercuspation and Centric Relation

**DOI:** 10.1155/2022/1439203

**Published:** 2022-01-19

**Authors:** Ilona Radej, Izabela Szarmach

**Affiliations:** Department of Orthodontics, Medical University of Białystok, ul. Waszyngtona 15a, 15-274 Białystok, Poland

## Abstract

**Purpose:**

This study is aimed at evaluating the impact of the craniofacial structure and occlusal conditions on the position of the articular heads of the mandibular condyles in the maximum intercuspal position (MIP) and comparing the centric relation (CR) and MIP of the mandibular condyles prior to orthodontic treatment.

**Methods:**

The studied group consisted of 33 women and 15 men (median age of 17.75 years). Contact points of opposing teeth in the MIP were assessed by hand-held casts. Condylar displacement (CD) in three spatial planes on both sides was measured on models mounted in an articulator using a mandibular position indicator (MPI). Patients were divided into groups according to craniofacial structures (vertical and horizontal growth directions). The Mann-Whitney, Kruskal-Wallis, post hoc Dwass-Steel-Critchlow-Fligner, and Pearson's *χ*^2^ independence tests as well as Spearman's nonparametric correlations were used in the statistical analyses.

**Results:**

Within the limitations of this study, no statistically significant correlation of CD with certain cephalometric measurements from a lateral cephalometric radiograph (ANB, SN-ML, and SGo/NMe) was observed. Correlation, however, was found between condylar displacement in the transverse axis and the mandibular plane angle SN-ML (*p* = 0.033) and also between condylar displacement in the anteroposterior axis and a midline shift of the mandible (*p* = 0.041). The results revealed a relationship between Angle's classification of molar position on the right side and anteroposterior CD values (*p* = 0.006).

**Conclusions:**

Cephalometric measurements cannot be used to predict CD at the level of the condyles. Analysis of occlusal conditions of models mounted in an articulator is desirable for patients with Angle's class I and lower jaw asymmetry.

## 1. Introduction

The centric relation (CR), which is defined as an optimal, orthopedically stable musculoskeletal position [1], provides maximum jaw stability and minimizes the force directed on each tooth during its functioning [2]. If occlusal interferences are present, then seating of the condyles in CR will be prevented and they will undergo a forced displacement as to allow for a stable occlusion of the teeth—the maximum intercuspal position (MIP). Such condylar displacement (CD) from CR to MIP occurs in 83.3% of the untreated population [3, 4]. Electromyographic studies suggest that the position of the articular heads of the mandibular condyles in the CR, when the teeth are in the MIP, allows for more harmonious and less intense work of the masticatory muscles [5] and provides a clinically repeatable reference position for developing a functional treatment plan for occlusion [6]. For this reason, the orthodontist should take this position into account in order to provide maximum comfort posttreatment for the patient. Orthodontic treatment should be planned with the intent of achieving maximal conformity of the condylar processes' positions in CR and MIP [2, 3, 7, 8].

It has been proposed that an articulator be used when planning orthodontic treatment to better assess the relationship between occlusion and position of the condyles [7, 9]. Mounting casts in an articulator can be helpful to better visualize conditions of occlusion in a stable musculoskeletal position and may offer the patient better quality treatment by providing a fuller diagnosis [10]. However, this approach is not always necessary. The majority of growing orthodontic patients usually end treatment before maturation of the temporomandibular joint (TMJ) has completed. An orthodontist's goal is to provide occlusal conditions within the patient's physiological tolerance or adaptability and should strive to achieve occlusal conditions resulting in a maximal stable musculoskeletal position. An articulator may be more helpful in adults due to completed TMJ growth and lower patient adaptability. This may be particularly helpful in patients with a hyperdivergent facial pattern. Ponces et al. [11] showed that the risk of making an incorrect diagnosis in this group of patients was about 30%. More studies with varying patient groups are needed. There is a lack of studies assessing in which patients mounting casts would be indicated, and many orthodontists are not willing to mount every single patient cast in an articulator because of the tediousness of this task. A meta-analysis of such studies would allow for a compromise in this discussion.

This cross-sectional study seeks to evaluate the impact of the craniofacial structure and occlusal conditions on the position of the mandibular condyles' articular heads in the MIP and compare the CR and MIP of the mandibular condyles prior to orthodontic treatment.

## 2. Materials and Methods

### 2.1. Patients

The studied group consisted of 48 patients (aged 11.50-50.30 yrs, median 17.75 yrs, 33 women and 15 men) with complete permanent dentition or interdental deficiencies due to premature loss of permanent teeth. Patients were recruited from the department of orthodontics of a university-based dental hospital. Gender was not a qualifying criterium for the study. Patients with facial trauma within the previous 5 years were excluded from the study. All patients were examined by the same experienced operator to avoid researcher-biased error.

### 2.2. Ethical Issues

The study was conducted according to the guidelines of the Helsinki Declaration of 1975, as revised in 2013, and approved by the Institutional Bioethics Committee of the Medical University of Białystok, protocol code number R-I-002/226/2017. Consent was given by all participants for a physical exam as well as for the usage/analysis of X-ray images and dental casts. Participants (or their legal guardians where applicable) signed appropriate consent forms before being enrolled in the study.

### 2.3. Methods

Interview and physical exam data were analyzed. Patients were divided into two groups using clinical indicators of temporomandibular disorders (TMDs) according to the Helkimo index [12]. One group included patients with no or minimal TMJ disorder (Helkimo Di0 and DiI) and the second group with more pronounced disorders (Helkimo DiII and DiIII).

A pantomographic and lateral telerentgenogram were taken of the head in the MIP with consideration of the Natural Head Position (Planmeca ProMax 3D Mid; Planmeca Oy, Helsinki, Finland), and high-quality orthodontic diagnostic casts were obtained from class IV synthetic dental stone (IV high-strength dental stone) (Fujirock EP; GC EUROPE N.V., Leuven, Belgium). Dental impressions were made with an irreversible hydrocolloid mass (Hydrogum 5; Zhermack S.p.A., Badia Polesine (Rovigo), Italy) on metal trays with deepened walls (Algilock; Hager & Werken GmbH & Co. KG, Duisburg, Germany). The analysis of diagnostic casts in the MIP was carried out on an intraoral record made of soft pink modelling wax (Modelling wax; Zhermack Sp. z o.o., Warszawa, Poland). The patient was asked to bite down on the wax in the MIP. The acquired impression's accuracy was rechecked in the patient's mouth after cooling in ice water. The CR was determined by the “power centric” method according to Roth [13] after prior neuromuscular deprogramming (pulsatile biting of a wooden spatula for 5-10 minutes) and then registering with wax (Bite Registration Sheet Wax, Almore International, Inc., Portland, OR, USA) on two pieces. A four-layer front wax record was obtained after positioning the patient at a 45° angle to the ground and heating the wax in a water bath to 57°C (06-DK-2000-1; Przedsiębiorstwo Techniczno-Handlowe “CHEMLAND,” Stargard, Poland). The patient's mandible was guided by the operator to avoid protrusion during the closing motion. The frontal impression was then cooled for 5 minutes in ice water and placed between the patient's dental arches together with the heated rear impression consisting of two or three layers. The patient's mandible was initially guided by the operator, and after reaching the appropriate grooves in the frontal impression, the patient was asked to bite down with increased force. Analysis of the casts in the CR was performed in a SAM 3 articulator. Registration of the maxilla's position using a face-bow (AxioQuick III, SAM Prazisionstechnik GmbH, München, Germany) allowed the upper cast to be mounted in the articulator using dental stone (Stodent III arti; Zhermack Sp. z o.o., Warszawa, Poland). CR registers were used to mount the mandibular cast. CR registration was repeated after 1-2 weeks in 10 randomly selected patients to assess the reproducibility of the CR records. These patients were fitted with new casts of the mandible to previously mounted casts of the maxilla. The results of both registrations underwent a comparative analysis.

Points of contacts of opposing teeth in CR and MIP were assessed. Contacts of opposing teeth in the MIP were assessed according to Angle's classification. The presence of a scissor-bite and cross-bite was verified. CD measurements on left and right sides were performed on casts using a mandibular position indicator (MPI) with a gauge (MPS, SAM Prazisionstechnik GmbH, München, Germany). Measurements were taken in three spatial planes assessing the positions of the condylar processes in the MIP in relation to the hinge axis of the articulator representing the CR. The difference was measured in the anteroposterior (*x*), vertical (*z*), and transverse (*y*) axes. The linear displacement of the position of the condylar processes in a given axis (Δ*x* and Δ*z*) was measured using graph paper and a magnifying glass with 0.1 mm measuring lines. Each measurement was performed twice, by the main researcher and by a second independent researcher, and then averaged. The device was recalibrated every 5 measurements using the MPI.

The condyle's position was assessed by criteria proposed by Utt et al. [14] and Hidaka et al. [15]. The ideal ranges of the position of the condylar process were accepted as *x* < 1, *z* < 1, and *y* < 0.5. Discrepancies of ≥2 mm in the anteroposterior or vertical axes or ≥0.5 mm in the transverse direction were considered clinically significant.

Cephalometric images were analyzed according to Jarabak and Björk's method in the Dolphin program (v1.8; Dolphin Imaging and Management Solutions, Chatsworth, California). Facial skeleton structure was assessed (rotation direction, mandibular plane inclination angle, and skeletal classes) and its impact on the CR-MIP difference. Patients were divided according to vertical cephalometric measurements into 3 groups depending on the SGo measurement (posterior face height) in relation to the NMe (anterior face height). Patients with ≤59% ratio were included in the hyperdivergent face type group. Normal divergent face types included those with a SGo/NMe (sella gonion/nasion menton) of 59-65. Patients with a ratio ≥ 65 were qualified to the hypodivergent group. Patients were also divided into 3 groups depending on the mandibular plane's inclination angle assessed by the NS/ML (nasion sella line-mandibular line) measurement according to Björk. A value of 33 ± 6 degrees was accepted as normal. Patients above this value were included in the posteriorotation group, and patients below this were included into the anteriorotation group.

Patients were also grouped according to horizontal cephalometric measurements to skeletal classes according to the ANB angle. Skeletal class I comprised an ANB (point A-nasion-point B) of 3.0 ± 2.5 degrees, class II included patients above this value, and class III included patients below this value.

### 2.4. Statistical Analysis

Quantitative variables were analyzed by nonparametric tests. Consistency of repeated measurements was assessed using intraclass correlation coefficients (ICCs) for absolute compliance. Comparisons between subgroups were performed using Mann-Whitney tests, while Kruskal-Wallis tests were used to compare larger subgroups, supplemented with post hoc tests according to Dwass-Steel-Critchlow-Fligner [16]. Relationships between pairs of quantitative variables were determined using Spearman's nonparametric correlation coefficients. Relationships between qualitative or ordinal variables were assessed by Pearson's *χ*^2^ independence tests. Calculations were made using IBM SPSS Statistics version 20.0. Statistical hypotheses were verified at a 0.05 significance level.

## 3. Results

**Figure 1:**
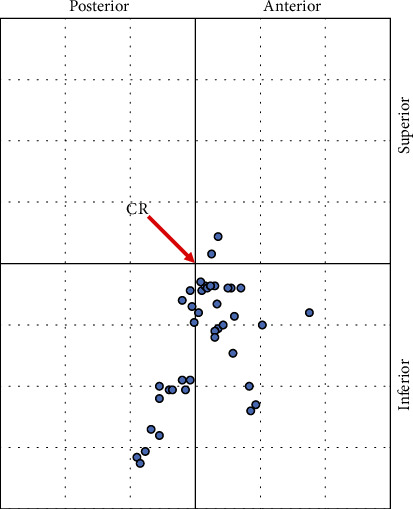
Graphical representation of displacement of condylar processes (*n* = 48). CR: centric relation.

The study included 48 patients. Characteristics of the studied group are presented in Tables 1–7.  Table 8 outlines the CD results with standard deviations in all three spatial planes. Most commonly observed displacements were downwards in the vertical axis, in the transverse axis, and to a lesser extent, in the horizontal axis.  Figure 1 presents the placement of the condylar process's position in the MIP in relation to CR on a 1 mm grid. Articular surfaces of the condylar processes were located in the inferoanterior range in 58.3% of patients (Δ*x* > 0 and Δ*z* > 0), which indicates displacement of the condylar process in the MIP down and forward. The condylar articular heads of the mandible were in the ideal range (Δ*x* < 1, Δ*z* < 1, and Δ*y* < 0.5) in 58.3% of patients.

Among 96 examined positions of the condyles of the mandible, significant CD in the vertical dimension (Δ*z* ≥ 2 mm) occurred in six instances (6.3%). Significant transverse CD (Δ*y* ≥ 0.5 mm) was registered also in six instances (6.3%). However, only 1 patient presented with a horizontal dimension of CD as Δ*x* ≤ −2 mm or Δ*x* ≥ 2 mm.

### 3.1. Assessment of the Test Method's Repeatability

Evaluation of the repeatability of the registrations after 1-2 weeks in 10 patients showed statistically significant agreement only in measurements of Δ*x* on the left side and Δ*z* on the right side (Table 9). Other measurements were not statistically significant. Negative Δ*z* values were recorded in 5 patients.

Due to the small number of negative Δ*z* measurements, these (negative) measurements were repeated. A patient was excluded from the study if the repeated result was positive. Repeat negative values were averaged and included in the statistical analysis.

### 3.2. Results of Statistical Analysis

Significant positive correlations of *Δx* and *Δz* with the corresponding measurements of the opposite side were observed (Table 10). Negative correlation of a bilaterally *Δx* shift with the left sided *Δz* measurement was also shown, meaning that the condylar shift backwards affects the left condyle causing a downward shift of its position. Positive correlation was also noted between the Δ*y* and *Δz* measurements on the right side, signifying that rightward condylar displacement affects the position of the right condyle, shifting it more downwards.

Displacement of the condylar process in the anteroposterior axis was associated with a mandibular midline displacement (*p* = 0.041). Protrusion of the left condylar process resulted in mandibular midline shift to the right (*p* = 0.029) Table 11.

The Pearson chi-square test for independence, which compared patients with an ideally positioned condylar process (Δ*x* < 1 mm, Δ*z* < 1 mm, and Δ*y* < 0.5 mm) with patients with CDs exceeding ideal values, did not show a significant correlation between CD and the Helkimo index and cephalometric measurements (ANB, SN-ML, and SGo/NMe). There was no statistically significant relationship between the range of condylar displacements in the 3 spatial planes and cephalometric variables (ANB and SGo/NMe) (data not shown). However, a relationship between the displacement of the condylar processes in the transverse axis and the mandibular plane inclination angle was observed (SN-ML) (*p* = 0.033). Patients with posteriorotation had more of a rightward CD, while those with anteriorotation presented with leftward CD (*p* = 0.022) Table 12. Correlation between the classification of occlusion of the first molars according to Angle on the right side and the anteroposterior CD (Δ*x*) was seen (*p* = 0.006) Table 13. A similar, but not statistically significant, trend was observed on the left side. The Dwass-Steel-Critchlow-Fligner test showed a statistically significant difference between Angle's classes I and II (*p* = 0.01) and II and III (*p* = 0.02). The condyles in the MIP were distal to the position in CR in Angle's class II, as opposed to class I where they were previously located anteriorly. There were no significant differences between classes I and III. No correlation between Angle's classes and CD in the *z* and *y* axes was seen. Furthermore, there was no relationship between CD size and the presence of scissor or cross-bite.

## 4. Discussion

The MPI used with the SAM articulator in assessing the condyles' positions proved to be accurate and reliable [3, 7, 17, 18]. Although most orthodontic patients could be assessed using ordinary hand-held casts, it is recommended to mount casts in an articulator since a malocclusion may mask the true maxilla-to-mandible ratio [13, 19, 20]. The occurrence of CD affects the orthodontic diagnostic process and changes malocclusion characteristics which are initially assessed in the MIP. In order to avoid errors in diagnosis and orthodontic treatment planning, lateral cephalometric images of the head should be converted from MIP to CR, especially when at least one axis displacement of ≥2 mm is present [19, 21].

Crawford [3] has shown that CDs larger than 1 mm in the horizontal or vertical planes or 0.5 mm in the transverse plane may adversely affect the TMJ. According to researchers, TMD symptomatology increases when condylar position indicator (CPI) measurements oscillate between 1 and 2 mm. CDs over 2.0 mm, in turn, are critical factors that should be taken into account when estimating the risk of a TMD. The orthodontist is unlikely to achieve as accurate CR and MIP compliance as the restorative dentist, but these studies suggest that the smaller the difference in the CR-MIP, the less likely TMD symptoms will develop. TMD is a multifactorial pathology, and a direct correlation between occlusion and TMD symptoms is difficult to determine. Nonetheless, the lack of scientific evidence is not a confirmation that such a relationship does not exist [22]. Orthodontic treatment routinely alters a patient's occlusion, and abnormal tooth contact is one of the potential risk factors for TMD; thus, orthodontists should provide the patient with a therapeutic position that minimizes this risk [2].

This study saw a much smaller percentage of patients with significant CD in all three directions compared to other studies [14, 15]. Significant displacement was observed in the transverse axis in 8 condyles (8.3%), in the vertical axis in 6 condyles (6.25%), and only in 1 condyle in the anteroposterior axis (1%). Five patients (10.4%) had significant displacement in the vertical or anteroposterior axis on at least one side. Seven (14.6%) patients had significant displacement in one of the three planes. Differences in the magnitude of shifts in the previous studies may have been due to anatomical differences of the TMJ's or dental arches, inclusion criteria of a given study, presence of a TMJ dysfunction, differences in the neuromuscular deprogramming methods, or differing CR registration techniques.

This research, as in other studies [3, 15, 17], noted mandibular condyles of most patients in the MIP were located in the anteroinferior or posteroinferior range. In a group of untreated patients, 95.8% of the condyles in the MIP were located below the CR, which also coincided with other studies [3]. In this study, the majority of condyles (58.3%) were located in an anteroinferior position, meaning that they were displaced anteroinferiorly. However, in other studies published previously [3, 11, 15, 17, 21, 23, 24], most condyles were located in the posteroinferior range. An anterior displacement is associated with interceptive occlusal contacts guiding the mandible in this direction. Ponces et al. [11] noted that in a group of hyper and normodivergent patients, as in most studies [3, 15, 21, 23, 24], rearward dislocation of the condyles was observed more frequently, whereas the condyles were displaced more forward in a group of hypodivergent patients. The forward displacements observed here are probably the result of the population's frequently encountered deeper facial structures. This may be associated with other muscular activity associated with varying facial patterns. Elevator muscles are stronger, placed more forward, and act more vertically in hypodivergent faces. This causes a greater release of force in the forward direction [3].

### 4.1. CD and Maxillofacial Morphology

This research confirms the results found in previous studies that there is no relationship between face morphology and CD size in anteroposterior and superoinferior measurements [14, 15]. However, a relationship between the displacement of the condylar processes in the transverse axis and the mandibular plane inclination angle (SN-ML) was observed in this study. This may be a result of altered masticatory muscle work in patients with improper vertical face shape. The data related to this topic are rare and contradictory, as such more in-depth studies are needed in this direction. Girardot [23] noticed larger vertical and anteroposterior CDs in hyperdivergent face morphologies, while Burke et al. [25] found a reduction in the upper joint space in the same type of face. Ponces et al. [11] showed that a group of patients with a hyperdivergent face type was characterized by a much greater CD along the vertical axis; however, horizontal CD occurred in these patients much less, the largest being in the group of hypodivergent patients. The studied group in this study consisted mostly of normodivergent and hypodivergent patients, commonly seen in a white population, and may have been the reason for the differing results. Lim et al. [26] showed that patients with a large CR-MIP discrepancy were characterized by specific facial features: decreased SNB angle, N Perpendicular to Pg, the height of the mandible's ramus, increased ANB angle, and inclination of the mandibular ramus in both CR and MIP. There were no significant differences in the measurement of the facial skeleton in the MIP in patients with small or large CR-MIP discrepancies, possible due to the fact that only patients with TMJ disc displacement and CR-MIP discrepancy were included in the study. The small number of patients with a significant CD in this study may have been insufficient to confirm this observation. However, considering anteroposterior disorders, Shildkraut et al. [21] showed that the differences in the position of the condylar processes of the mandible in the vertical dimension Δ*z* occurred equally in patients with skeletal classes I and II. The differing results of individual studies may result from varying research methodologies or nonuniform qualification of patients such as the inclusion or elimination of patients with TMD symptoms. The neuromuscular system can respond to occlusal interferences in two ways: one by moving the condyle in the joint to achieve maximum occlusive contacts, while the second results in the appearance of an anterior open bite and contacts only on the lateral teeth. In the second situation the CD is reduced. Another factor affecting the heterogeneity of tests is the exclusion of negative values of vertical axis Δ*z* displacements, commonly resulting from an error at the stage of obtaining the CR registration. Negative Δ*z* values should not occur in patients without TMD. However, this may occur when patients with symptoms of TMJ degeneration are included in a study. Varying research results and the small number of such studies in Europe and the lack there of in Poland show the need to continue these types of projects on a larger scale, taking into account the same number of patients with different facial morphologies.

### 4.2. CD Asymmetry

This study saw a mandibular midline shift in 54.2% of patients, although bilateral Δ*x* measurements showed a significant relationship (nonparametric Spearman correlation 0.658). Measurements of Δ*z* were also significantly correlated (nonparametric Spearman correlation 0.609). Some asymmetry was observed due to the fact that this correlation was not perfect. In this study, when the CD was downwards, it was greater on the left side, as was in the study by Hidaka et al. [15]. The left condyle moved forward in the MIP (median shift of 0.25 mm), while the right condyle showed almost no tendency to move (median shift of 0.03 mm). This asymmetry resulted in rightward midline shift of the mandible in the MIP. Statistical analysis confirmed this observation to be significant (*p* = 0.029). This study also noticed a negative correlation between anteroposterior and superoinferior measurements, admittedly only concerning the left condyle. Displacement of the left condyle forward affects its upward position. Hidaka et al. [15] also noticed pronounced asymmetry of the CD, noting that displacement downwards was larger on the left while forward displacement was larger on the right. These features may cause displacement of the anterior portion of the mandible to the left, however, Hidaka et al. showed a weak positive correlation in this direction. Studies by Pullinger et al. [27] have also shown a low occurrence of lateral shifts and a slight association with asymmetry of right and left condyle positions. Therefore, mandibular condyle displacement can be one of many components of mandibular asymmetry. The clinical implications resulting from these observations should prompt in-depth investigations, especially in patients with midline displacement of the mandibular arch. When malocclusion is due to asymmetry of condyles in their articular fossa, the CR should improve the occlusal condition. This may allow a skeletal component of the defect to be excluded from the diagnosis and correction of the midline through teeth movements may not be necessary to the extent that this would have been planned in the MIP. However, this study did not confirm a relationship between the presence of transverse malocclusions and CD size. This may be due to the small number of patients included in the study with this particular type of defect.

### 4.3. CD and Angle's Classification

Wood and Elliot [28] noticed that the mandibular body and teeth can dislocate distally, resulting in an increased horizontal overlap, reduced vertical overlap, and a change in molar relation from Angle's classes I to II. This research confirms these observations at the level of the condylar processes. The median anterior shift of Δ*x* = 0.3 mm in patients with Angle's class I explains the occurrence of Angle's class II on casts registered in CR, after when reaching first contact in class II may produce an anterior shift and final contact is achieved in the MIP in class I. Following this line of reasoning, a median shift of Δ*x* = 0.33 mm in Angle class III could reduce the severity of the defect on CR-registered casts. However, this study did not find a significance correlation here. This may be explained by the small number of patients with class III. The median displacement of Δ*x* = −0.3 in patients of Angle's class II after mounting casts in CR could also reduce the defect, which changes due to the predisposition to shift posteriorly in order to achieve an MIP. Significant variations in Δ*x* in classes II and III (*p* = 0.02) confirm this theory. However, according to Lim et al. [26], patients with high CR-MIP displacement at the level of the incisors in CR have a more retracted mandible and a more vertical growth pattern, which may exacerbate the severity of a class II defect. This tendency is mainly seen in patients with a vertical growth pattern, hence resulting in the discrepancies found in this study. No statistically significant relationship was found between the molar classification according to Angle on the left side and the value of Δ*x* (data not shown). This is most likely due to the small study group. A similar tendency, similar to that seen on the right side, however, suggests that with a larger group, this result would probably be significant. However, Utt et al. [14] found no differences in the size of MPI measurements between patients from classes I and II; therefore, further research is required to confirm these observations. Nonetheless, particular attention should be paid to the need for CR registration in patients with Angle's class I where the condyles in the MIP are often displaced anteriorly.

### 4.4. Potential Research Errors

A repeated recording of the CR performed to assess measurement error after 1-2 weeks did not reveal statistically significant compliance of most measurements. This is probably because the “power centric” method was developed to record condylar position on the day of registration. The method's repeatability has been previously documented [13, 29, 30]. However, in the event of occlusive interference, an incorrect mandibular closing pattern may persist in order to avoid excessive occlusive forces on the teeth. This situation may impede determination of the CR [13, 19, 31]. An ideal study protocol would require complete deprogramming of all patients by splint therapy. However, this is not practical in small-scale studies due to long-term splint therapy. Including such deprogramming in future studies could increase their value.

Some sources suggest excluding negative Δ*z* values [13]. This study saw low values of negative Δ*z* measurements; thus, the measurements were repeated in these patients. If the repeated result was again negative, it was included in the study and remaining patients were rejected. Negative values may have resulted from the use of an averaged hinge axis in the articulator, insufficient patient deprogramming, excessive muscle tone/force, or TMJ degeneration. Slavicek attributes this finding to a compression phenomenon [19]. The risk of error was minimized due calibration of the apparatus every five patients as well as measurements being performed by two independent researchers.

This study qualifies as an early attempt to assess the impact of the craniofacial structure and occlusal conditions on the position of the mandibular condyles. More studies with a larger patient base are needed. Studies to date have focused on hyperdivergent patients where this study involved hypodivergent ones which allowed for the observation of differences in the position of the condylar processes in Angle's classes I and II and the correlation between lateral shifts of the mandible and an asymmetry of right and left condyle positions. A larger number of similar studies would allow for a meta-analysis to be carried out, which would help orthodontists assess the need for cast articulation in orthodontic treatment in various patient groups.

## 5. Conclusion

With the limitation of the present study, cephalometric measurements (ANB, Sgo/NMe, and SN-ML) do not provide sufficient information to predict the frequency, size, and direction of CD at the level of the condylar processes. Cast analysis in an articulator makes it possible to diagnose the size and direction of the CD and is particularly desirable in patients with Angle class I, in whom an anterior CD may mask the occurrence of an Angle class II in CR. In addition, it would allow an assessment of whether the malocclusion is the result of an eccentric shift of the mandible, in which the asymmetrical displacement of the condyles results in a mandibular midline shift.

## Figures and Tables

**Table 1:** Characteristics of study group, *n* = 48.

	Average ± SD	Median [Min, Max]
Age (yrs)	21.91 ± 9.01	17.75 [11.50, 50.30]
NS/ML (degrees)	31.21 ± 5.94	30.75 [19.20, 47.90]
ANB (degrees)	2.48 ± 2.62	2.65 [-4.10, 7.00]
SGo/NMe (%)	67.98 ± 4.49	67.90 [57.10, 82.40]

NS/ML: nasion sella line/mandibular line; ANB: A point-nasion-B point; SGo/NMe: sella gonion/nasion menton; SD: standard deviation.

**Table 2:** Skeletal class categories—ANB angle (degrees).

	*n* (%)
≤0.5 (class III)	12 (25.0)
0.5-5.5 (class I)	30 (62.5)
≥5.5 (class II)	6 (12.5)

Total	48 (100.0)

ANB: A point-nasion-B point.

**Table 3:** SGo/NMe categories (%).

	*n* (%)
≤59	1 (2.1)
59-65	10 (20.8)
≥65	37 (77.1)

Total	48 (100.0)

SGo/NMe: sella gonion/nasion menton.

**Table 4:** NS/ML categories (degrees).

	*n* (%)
≤27	14 (29.2)
27-39	29 (60.4)
≥39	5 (10.4)

Total	48 (100.0)

NS/ML: nasion sella line/mandibular line.

**Table 5:** Helkimo categories.

	*n* (%)
0 + 1	6 (12.5)
2 + 3	42 (87.5)

Total	48 (100.0)

**Table 6:** Angle's classification: left and right (L/R).

		Left *n* (%)	Right *n* %
	I	20 (41.7)	22 (45.8)
	II	7 (14.6)	8 (16.7)
	II (1/2 unit)	7 (14.6)	4 (8.3)
	III	1 (2.1)	2 (4.2)
	III (1/2 unit)	9 (18.8)	9 (18.8)
	Total	44 (91.7)	45 (93.8)
No data	No assessment available	4 (8.3)	3 (6.3)

Total		48 (100.0)	48 (100.0)

**Table 7:** Presence of cross-bite or lingual occlusion.

	Cross-bite				Lingual occlusion	
	Incisors *n* (%)	Cuspids *n* (%)	Premolars *n* (%)	Molars *n* (%)	Premolars *n* (%)	Molars *n* (%)
No	46 (95.8)	44 (91.7)	40 (83.3)	44 (91.7)	46 (95.8)	48 (100.0)
Yes	2 (4.2)	4 (8.3)	8 (16.7)	4 (8.3)	2 (4.2)	0 (0)

Total	48 (100.0)	48 (100.0)	48 (100.0)	48 (100.0)	48 (100.0)	48 (100.0)

**Table 8:** Displacement of condylar processes in the maximal intercuspal position (in mm), *n* = 48.

	Average ± SD	Median [Min, Max]
Δ*x* R	0.03 ± 0.6	0.03 [-1.2, 1.2]
Δ*x* L	0.27 ± 0.68	0.25 [-1.2, 2.3]
Δ*y*	−0.01 ± 0.31	-0.02 [-0.6, 1.25]
Δ*z* R	0.64 ± 0.8	0.45 [-0.6, 3.5]
Δ*z* L	0.88 ± 0.7	0.7 [-0.15, 3.5]
Δ*x*	0.15 ± 0.59	0.18 [-0.9, 1.75]
Δ*z*	0.76 ± 0.69	0.54 [-0.23, 3.5]

Δ*x*: condylar displacement in anteroposterior axis; Δ*y*: condylar displacement in vertical axis; Δ*z*: condylar displacement in transverse axis; R: right side; L: left side; SD: standard deviation.

**Table 9:** Intraclass correlation coefficient (ICC) (2.1) for absolute compliance for a single measurement.

	Intraclass correlation for absolute agreement for a single measurement	
	ICC (2.1)	*p*
Δ*x* R	0.04	0.436
Δ*x* L	0.59	0.022
Δ*y*	0.49	0.072
Δ*z* R	0.65	0.012
Δ*z* L	0.31	0.189
Δ*x*	0.30	0.133
Δ*z*	0.41	0.104

Δ*x*: condylar displacement in anteroposterior axis; Δ*y*: condylar displacement in vertical axis; Δ*z*: condylar displacement in transverse axis; R: right side; L: left side.

**Table 10:** Spearman nonparametric correlations between measurements.

	Age	Δ*x*_R	Δ*x*_L	Δ*z*_R	Δ*z*_L	Δ*y*
*Δx*_P	-0.097					
*Δx*_L	-0.235	0.658^∗∗^				
*Δz*_P	-0.028	-0.084	-0.098			
*Δz*_L	-0.046	-0.289^∗^	-0.325^∗^	0.609^∗∗^		
Δ*y*	-0.023	0.119	0.261	0.324^∗^	0.160	
ANB	-0.099	-0.036	-0.118	0.028	0.047	0.155
SGo/NMe	0.172	0.016	-0.062	-0.119	-0.165	-0.176
NS/ML	-0.184	0.060	0.085	0.182	0.252	0.226

^∗∗^ *p* < 0.01 ^∗^*p* < 0.05						

Δ*x*: condylar displacement in anteroposterior axis; Δ*z*: condylar displacement in transverse axis;  Δ*y*: condylar displacement in vertical axis; R: right side; L: left side; ANB: A point-nasion-B point; SGo/NMe: sella gonion/nasion menton; NS/ML: nasion sella line/mandibular line.

**Table 11:** Comparison of the mandibular midline shift on the condylar processes (in mm).

Midline of mandible		Δ*x*_R	Δ*x*_L	Δ*y*	Δ*z*_R	Δ*z*_L	Δ*x*	Δ*z*
Compliant with the face midline [0] *n* = 22	Average ± SD	−0.01 ± 0.59	0.02 ± 0.60	−0.04 ± 0.22	0.75 ± 0.90	0.96 ± 0.79	0.00 ± 0.53	0.86 ± 0.79
	Median [Min, Max]	0.00 [-1.15, 1.05]	-0.10 [-1.20, 1.20]	-0.03 [-0.50, 0.35]	0.55 [-0.60, 3.50]	0.98 [-0.15, 3.50]	0.08 [-0.90, 1.13]	0.95 [-0.23, 3.50]

Right shift [1] *n* = 16	Average ± SD	0.18 ± 0.50	0.58 ± 0.67	-0.07 ± 0.30	0.36 ± 0.56	0.55 ± 0.24	0.38 ± 0.54	0.45 ± 0.33
	Median [Min, Max]	0.15 [-0.60, 1.20]	0.60 [-0.50, 2.30]	-0.09 [-0.60, 0.78]	0.13 [-0.20, 1.75]	0.60 [0.05, 1.00]	0.33 [-0.55, 1.75]	0.40 [-0.08, 1.2]

Left shift [2] *n* = 10	Average ± SD	−0.11 ± 0.77	0.35 ± 0.69	0.18 ± 0.44	0.86 ± 0.82	1.23 ± 0.81	0.12 ± 0.71	1.04 ± 0.76
	Median [Min, Max]	-0.03 [-1.20, 1.15]	0.45 [-0.70, 1.15]	0.07 [-0.30, 1.25]	0.63 [0.00, 2.40]	1.10 [0.30, 2.60]	0.25 [-0.85, 1.15]	0.83 [0.15, 2.50]

*p* (Kruskal-Wallis test)		0.570	**0.041**	0.208	0.115	0.067	0.169	0.121

*p* (Dwass-Steel-Critchlow-Fligner test)	[0] vs. [1]	0.667	**0.029**	0.748	0.172	0.233		
	[0] vs. [2]	0.921	0.394	0.327	0.956	0.718		
	[1] vs. [2]	0.642	0.870	0.253	0.178	0.056		

Δ*x*: condylar displacement in anteroposterior axis; Δ*z*: condylar displacement in transverse axis; Δ*y*: condylar displacement in vertical axis; R: right side; L: left side; SD: standard deviation.

**Table 12:** Displacement of the condylar processes depending on the SN/ML angle measurement (in mm).

SN/ML (degrees)		Δ*y*	Δ*x*	Δ*z*
≤27 *n* = 14	Average ± SD	−0.13 ± 0.20	0.18 ± 0.58	0.58 ± 0.47
	Median [Min, Max]	-0.10 [-0.48, 0.15]	0.16 [-0.80, 1.15]	0.44 [0.05, 1.43]

27-39 *n* = 29	Average ± SD	0.00 ± 0.34	0.06 ± 0.58	0.75 ± 0.62
	Median [Min, Max]	-0.02 [-0.60, 1.25]	0.10 [-0.90, 1.75]	0.60 [-0.23, 2.50]

≥39 *n* = 5	Average ± SD	0.26 ± 0.31	0.64 ± 0.49	1.32 ± 1.29
	Median [Min, Max]	0.20 [-0.02, 0.78]	0.85 [-0.08, 1.13]	1.15 [0.23, 3.50]

*p* (Kruskal-Wallis test)		**0.033**	0.115	0.297

*p* (Dwass-Steel-Critchlow-Fligner test)	≤27 vs. 27-39	0.456		
	≤27 vs. ≥39	**0.022**		
	27-39 vs. ≥39	0.125		

Δ*x*: condylar displacement in anteroposterior axis; Δ*z*: condylar displacement in transverse axis; Δ*y*: condylar displacement in vertical axis; NS/ML: nasion sella line/mandibular line; SD: standard deviation.

**Table 13:** Displacement of the condylar processes depending on the Angle classification (in mm) on the right side.

Angle's classification		Δ*x*	Δ*y*	Δ*z*
I *n* = 22	Average ± SD	0.35 ± 0.60	0.04 ± 0.39	0.65 ± 0.62
	Median [Min, Max]	0.30 [-0.85, 1.75]	0.00 [-0.60, 1.25]	0.46 [-0.08, 2.50]

II *n* = 12	Average ± SD	−0.26 ± 0.39	−0.08 ± 0.15	0.79 ± 0.41
	Median [Min, Max]	-0.30 [-0.80, 0.43]	-0.04 [-0.38, 0.18]	0.83 [0.23, 1.40]

III *n* = 11	Average ± SD	0.31 ± 0.52	−0.07 ± 0.30	0.96 ± 1.01
	Median [Min, Max]	0.33 [-0.78, 1.13]	-0.20 [-0.50, 0.45]	0.48 [0.15, 3.50]

*p* ^∗∗^		**0.006**	0.567	0.412

*p* ^∗∗∗^	I vs. II	**0.010**	0.569	0.337
	I vs. III	0.987	0.759	0.863
	II vs. III	**0.020**	1.000	0.903

Δ*x*: condylar displacement in anteroposterior axis; Δ*z*: condylar displacement in transverse axis; Δ*y*: condylar displacement in vertical axis; SD: standard deviation.

## Data Availability

The datasets used and/or analyzed during the current study are available from the corresponding author on reasonable request.

## References

[1] Ferro K. J., Morgano S. M., Driscoll C. F. (2017). The Glossary of Prosthodontic Terms: Ninth Edition. *The Journal of Prosthetic Dentistry*.

[2] Okeson J. P. (2015). Evolution of occlusion and temporomandibular disorder in orthodontics: Past, present, and future. *American Journal of Orthodontics and Dentofacial Orthopedics*.

[3] Crawford S. D. (1999). Condylar axis position, as determined by the occlusion and measured by the CPI instrument, and signs and symptoms of temporomandibular dysfunction. *The Angle Orthodontist*.

[4] Hodge L. C., Mahan P. E. (1967). A study of mandibular movement from centric occlusion to maximum intercuspation. *The Journal of Prosthetic Dentistry*.

[5] Williamson E. H., Lundquist D. O. (1983). Anterior guidance: its effect on electromyographic activity of the temporal and masseter muscles. *The Journal of Prosthetic Dentistry*.

[6] Wiens J. P., Goldstein G. R., Andrawis M., Choi M., Priebe J. W. (2018). Defining centric relation. *The Journal of Prosthetic Dentistry*.

[7] Roth R. H. (1973). Temporomandibular pain-dysfunction and occlusal relationships. *The Angle Orthodontist*.

[8] Dawson P. E. (1995). New definition for relating occlusion to varying conditions of the temporomandibular joint. *The Journal of Prosthetic Dentistry*.

[9] Roth R. H. (1976). The maintenance system and occlusal dynamics. *Dental Clinics of North America*.

[10] Martin D., Cocconi R. (2012). Orthodontic dental casts: the case for routine articulator mounting. *American Journal of Orthodontics and Dentofacial Orthopedics*.

[11] Ponces M. J., Tavares J. P., Lopes J. D., Ferreira A. P. (2014). Comparison of condylar displacement between three biotypological facial groups by using mounted models and a mandibular position indicator. *The Korean Journal of Orthodontics*.

[12] Helkimo M. (1974). Studies on function and dysfunction of the masticatory system. II. Index for anamnestic and clinical dysfunction and occlusal state. *Svensk Tandlakare tidskrift. Swedish Dental Journal*.

[13] Roth R. H. (1981). Functional occlusion for the orthodontist. *Journal of Clinical Orthodontics*.

[14] Utt T. W., Meyers C. E., Wierzba T. F., Hondrum S. O. (1995). A three-dimensional comparison of condylar position changes between centric relation and centric occlusion using the mandibular position indicator. *American Journal of Orthodontics and Dentofacial Orthopedics*.

[15] Hidaka O., Adachi S., Takada K. (2002). The difference in condylar position between centric relation and centric occlusion in pretreatment Japanese orthodontic patients. *The Angle Orthodontist*.

[16] Hollander M., Wolfe D. (1999). *Nonparametric Statistical Methods*.

[17] Wood D. P., Korne P. H. (1992). Estimated and true hinge axis: a comparison of condylar displacements. *The Angle Orthodontist*.

[18] Alexander S. R., Moore R. N., DuBois L. M. (1993). Mandibular condyle position: comparison of articulator mountings and magnetic resonance imaging. *American Journal of Orthodontics and Dentofacial Orthopedics*.

[19] Slavicek R. (1988). Clinical and instrumental functional analysis and treatment planning. Part 4. Instrumental analysis of mandibular casts using the mandibular position indicator. *Journal of Clinical Orthodontics*.

[20] Parker W. S. (1978). Centric relation and centric occlusion--an orthodontic responsibility. *American Journal of Orthodontics*.

[21] Shildkraut M., Wood D. P., Hunter W. S. (1994). The CR-CO discrepancy and its effect on cephalometric measurements. *The Angle Orthodontist*.

[22] Michelotti A., Iodice G. (2010). The role of orthodontics in temporomandibular disorders. *Journal of Oral Rehabilitation*.

[23] Girardot R. A. (2001). Comparison of condylar position in hyperdivergent and hypodivergent facial skeletal types. *The Angle Orthodontist*.

[24] Karl P. J., Foley T. F. (1999). The use of a deprogramming appliance to obtain centric relation records. *The Angle Orthodontist*.

[25] Burke G., Major P., Glover K., Prasad N. (1998). Correlations between condylar characteristics and facial morphology in class II preadolescent patients. *American Journal of Orthodontics and Dentofacial Orthopedics*.

[26] Lim W. H., Choi B., Lee J. Y., Ahn S. J. (2014). Dentofacial characteristics in orthodontic patients with centric relation-maximum intercuspation discrepancy. *The Angle Orthodontist*.

[27] Pullinger A. G., Solberg W. K., Hollender L., Petersson A. (1987). Relationship of mandibular condylar position to dental occlusion factors in an asymptomatic population. *American Journal of Orthodontics and Dentofacial Orthopedics*.

[28] Wood D. P., Elliott R. W. (1994). Reproducibility of the centric relation bite registration technique. *The Angle Orthodontist*.

[29] Wood D. P., Floreani K. J., Galil K. A., Teteruck W. R. (1994). The effect of incisal bite force on condylar seating. *The Angle Orthodontist*.

[30] Holen Galeković N., Fugošić V., Braut V., Ćelić R. (2017). Reproducibility of centric relation techniques by means of condyle position analysis. *Acta Stomatologica Croatica*.

[31] Dawson P. E. (1979). Centric relation. Its effect on occluso-muscle harmony. *Dental Clinics of North America*.

